# On the Temporal Dynamics of Tool Use

**DOI:** 10.3389/fnhum.2020.579378

**Published:** 2020-12-07

**Authors:** François Osiurak, Giovanni Federico, Maria A. Brandimonte, Emanuelle Reynaud, Mathieu Lesourd

**Affiliations:** ^1^Laboratoire d'Etude des Mécanismes Cognitifs, Université de Lyon, Lyon, France; ^2^Institut Universitaire de France, Paris, France; ^3^Laboratory of Experimental Psychology, Suor Orsola Benincasa University, Naples, Italy; ^4^Laboratoire de Psychologie, Université de Bourgogne Franche-Comté, Besançon, France

**Keywords:** tool use, affordance, embodied cognition, technical reasoning, motor control

## Introduction

We humans have a proclivity for materiality as evidenced by our ability to use and make tools or build constructions. Over generations, this proclivity has led to considerably modify the surface of the Earth, a phenomenon known as cumulative technological culture (Osiurak and Reynaud, 2020). This is a fact: we are nowadays surrounded by artifacts (defined here as tools made for a specific purpose). In this context, the epistemological belief can emerge that humans have become mere manipulators, much more concerned with how to manipulate artifacts to make them work than to understand how they work (Osiurak et al., 2020). At a neurocognitive level, this belief has led to the hypothesis that the human brain has developed adaptive mechanisms enabling the selection and planning of the appropriate motor actions^1^ to manipulate artifacts (Heilman et al., 1982; Rothi et al., 1991; Buxbaum, 2001; van Elk et al., 2014). Thus, when we see an artifact, we might automatically activate not only the motor representation of how to grasp it (so-called structural affordance; e.g., a power grip to grasp and move a hammer from one location to another) but also the motor representation of how to use it in a functional way^2^ (so-called functional affordance; e.g., a power grip and a broad oscillation of the elbow joint to grasp and use a hammer to pound a nail; Buxbaum and Kalénine, 2010; Thill et al., 2013; see also Bach et al., 2014; Kourtis and Vingerhoets, 2015; Kourtis et al., 2018). This is the automatic activation hypothesis of functional affordances^3^. In this Opinion article, we question the theoretical and empirical validity of the strong form of this hypothesis and propose an alternative view derived from the technical reasoning hypothesis and based on the idea that technical reasoning and motor control interact together via a cascade mechanism. Understanding how individuals are able to use tools necessarily requires discussing findings from both behavioral and brain-related studies (e.g., neurophysiology, neuroimaging). In this article, we will focus on behavioral findings, suggesting that the discussion initiated here is incomplete and would need to be extended to brain-related findings.

## The Strong Form of the Automatic Activation Hypothesis of Functional Affordances

**Figure 1:**
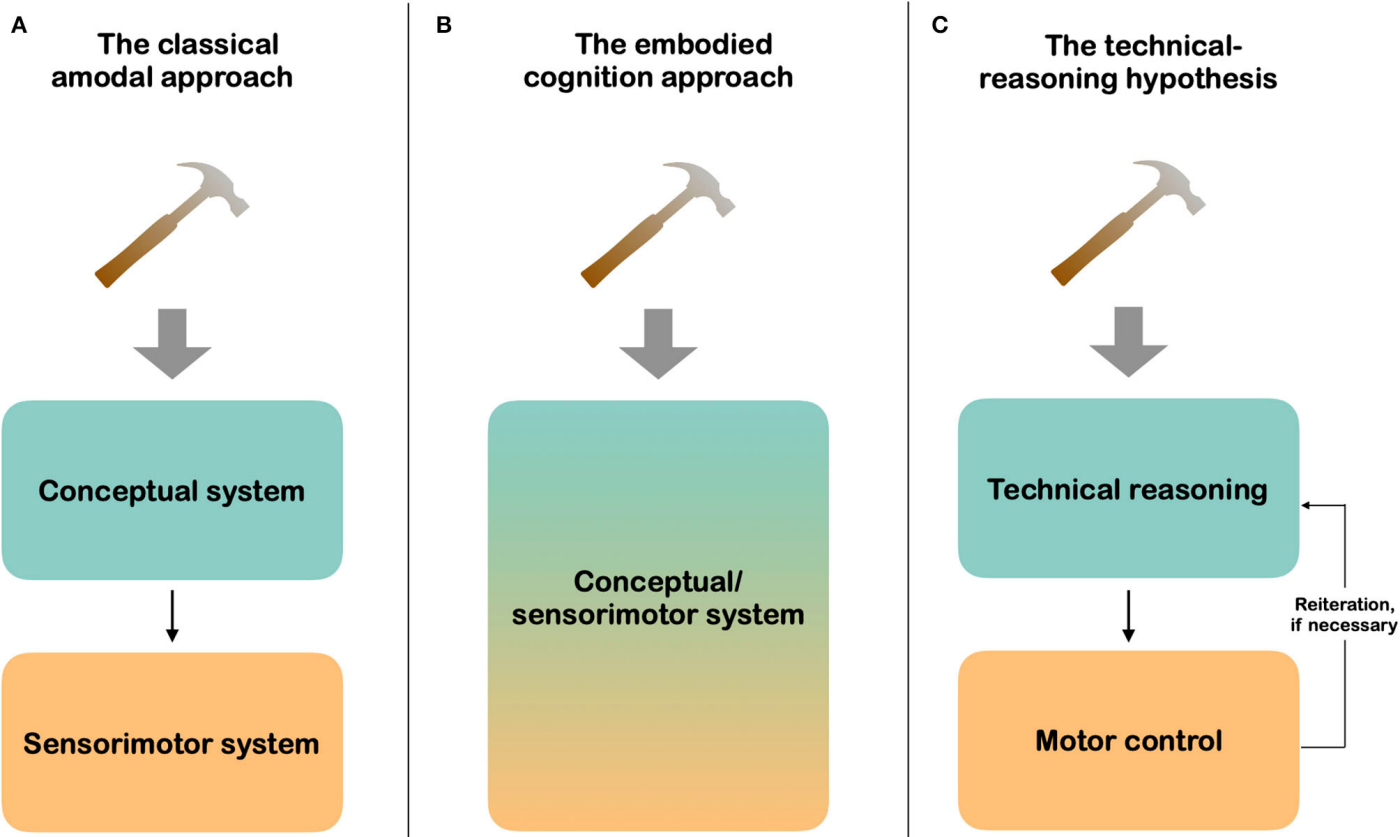
Cognitive approaches to tool use. As shown in **(A,B)** the initial project of the embodied cognition approach was to propose an alternative to the classical amodal approach in assuming that tool use is not based on a cascade mechanism “conceptual system ⇒ sensorimotor system” but on the automatic activation of functional affordances within a unique conceptual/sensorimotor system. As explained in the present article, **(C)** the cascade mechanism through which technical reasoning and motor control interact is one of the key assumptions of the technical reasoning hypothesis, which also posits that this mechanism is reiterative, if necessary.

The automatic activation hypothesis of functional affordances can be viewed as a strong form of the embodied cognition approach to tool use^4^. The embodied cognition approach has emerged in contradiction to the classical amodal approach, which considers that our understanding of the physical world is conceived in an abstract way (Fodor, 1975; see also Mahon and Caramazza, 2008; Mahon, 2015). In this classical amodal approach, the conceptual level is distinguished from the sensorimotor level: we conceive our actions first, and then we decide which motor actions to perform (Figure 1A). By contrast, the embodied cognition approach assumes that the conceptual level is grounded in our sensorimotor systems (Figure 1B). Thus, conceiving an action with an artifact consists of simulating the interactions we have had with it at the sensorimotor level (Barsalou, 2008, 2009; Mizelle and Wheaton, 2010; Thill et al., 2013). This approach in its strong form *inevitably* leads to considering that seeing an artifact automatically activates the motor representations associated with *its usual function*, namely functional affordances (e.g., Rothi et al., 1991; Buxbaum, 2001; Bach et al., 2014; van Elk et al., 2014). We emphasize the word “inevitably” because this automatic activation hypothesis is the only way of making a clear difference between the classical amodal approach and the embodied cognition approach. Indeed, if these motor representations can be activated via a kind of cascade mechanism involving non-sensorimotor representations at earlier stages, then this amounts to proposing the same framework as that of the classical amodal approach (i.e., conceptual level ⇒ sensorimotor level). Therefore, these motor representations must be activated without requiring additional cognitive processes, hence the automatic activation hypothesis. We emphasize the phrase “its usual function” because the embodied cognition approach aims to propose an alternative way of considering the conceptual level (i.e., sensorimotor, not abstract). In this respect, this approach does not aim to explain how our motor-control system can perform non-tool-related motor actions, such as grasping or reaching because otherwise this would not be an alternative but rather a complementary approach to the classical amodal approach. In other words, the interest of the embodied cognition approach precisely lies in assuming that motor representations are involved at the conceptual level in order to specify how to use artifacts according to their usual function^5^.

## The Orientation Effect

The idea that functional affordances are automatically activated by the sight of artifacts is more an assumption than a hypothesis for embodied cognition models of tool use. Put differently, this assumption has generated a great number of interesting findings. However, much less empirical effort has been spent on investigating its validity. Key evidence may come from the orientation effect initially reported by Tucker and Ellis (1998). In their seminal article, they presented participants with pictures of artifacts with the handle oriented toward either the right or the left. The orientation of the handle was not relevant to the task, which consisted in judging the vertical orientation (upright/inverted). Yet, participants were faster to respond with a right-hand keypress when the handle was oriented to the right and vice versa. Tucker and Ellis (1998) did not interpret this orientation effect as evidence for an automatic activation of functional affordances, but of motor affordances, without specifying clearly whether it may concern structural affordance, functional affordances, or both. Later, they stated that the orientation effect (i.e., the mere presence of faster keypress responses when the handle is oriented to the same side) was far more consistent with a structural rather than a functional affordance interpretation (Symes et al., 2007; see also Vingerhoets et al., 2009). Hence, there appears to be a gap between reporting this so-called orientation effect and demonstrating that seeing an artifact automatically activates the motor representation of how to use it for its usual function (i.e., functional affordances). However, as discussed above, the main problem is that, if this orientation effect only concerns structural affordances and, as a result, is not specific to tool-use actions, then it does not provide any support for the embodied cognition approach. In addition, the generalization and robustness of this orientation effect have been subject to an intense debate (e.g., for conflicting results, Anderson et al., 2002; Cho and Proctor, 2010, 2011, 2013; Matheson and Thompson-Schill, 2019; Pellicano et al., 2019; Kostov and Janyan, in press; Pellicano and Binkofski, in press; for a review, see Osiurak and Badets, 2016; Azaad et al., 2019). To sum up, this orientation effect at best reflects the automatic activation of structural affordances but not of functional affordances. Yet, many studies have capitalized on it to develop a strong form of the embodied cognition approach to tool use, organized around the automatic activation of functional affordances (e.g., Creem and Proffitt, 2001; Grèzes et al., 2003; Buxbaum and Kalénine, 2010; Bach et al., 2014; van Elk et al., 2014; Kourtis and Vingerhoets, 2015; Kourtis et al., 2018).

## From Theoretical Issues

The automatic activation hypothesis of functional affordances also presents other limitations. We have also emphasized that this hypothesis is not economic at a motor level or that it does not explain what happens when the tool-use action is not unimanual but bimanual (Osiurak et al., 2011; Osiurak and Badets, 2016). Here, we will limit our discussion to another theoretical limitation, which concerns the temporal dynamics of the so-called automatic activation, namely, an aspect that is rarely addressed by the proponents of the embodied cognition approach (but see Bub et al., 2018; see also Trumpp et al., 2014). The outstanding question is how these functional affordances activate. Let's elaborate on it to answer this question.

A first possibility is that looking at the manipulative part of an artifact (e.g., a handle) is sufficient to activate the associated functional affordance. This possibility is unlikely. Indeed, looking at the manipulative part, say the handle, is not enough to determine what is the usual function of the artifact. At best, we might automatically activate some structural affordances (e.g., grasping or reaching actions) based on the physical properties of the handle. In addition, it is impossible to activate the appropriate functional affordances by merely looking at the handle of the artifact because the same handle can be associated with a multitude of different artifacts with different uses. To solve this issue, a second possibility is that functional affordances are automatically activated by the sight of the active part of the artifact (e.g., the head of the hammer). However, this raises another theoretical issue. Functional affordances are thought to specify the kinematic and postural parameters associated with the motor action useful to use the artifact for its usual function (e.g., van Elk et al., 2014). These parameters are derived from the experience we have with artifacts and directly targets the manipulative part, namely, the contact zone between the user and the artifact. Therefore, it appears impossible to envisage that functional affordances are automatically activated only by looking at the functional part. They must also be activated after looking at the manipulative part. In other words, the automatic activation of this functional affordances necessarily requires an exploratory gaze pattern consisting in looking at the functional part, first, and at the manipulative part, second. This is nevertheless not predicted by the automatic activation hypothesis of functional affordances. This also raises the theoretical question of when the functional affordances are activated. For instance, are they activated just after looking at the functional part or rather after looking at the manipulative part? What if the functional part does not seem appropriate for the current purpose? Do we nevertheless pursue the procedure of activating the functional affordances associated with the artifact?

## To Empirical Counterarguments

The idea that people focus first on the active part of a tool/artifact has been supported by a significant body of evidence. For instance, van Elk et al. (2008) presented participants with pictures of a model using artifacts and asked them to detect whether the goal location was correct (i.e., whether the active part was oriented toward the correct part of the model) or whether the grip was correct (i.e., whether the manipulative part was correctly grasped) They found that the detection of the correctness of the goal location was faster than the detection of the correctness of the grip. Other studies have corroborated this pattern of results in an observational context (e.g., Massen and Prinz, 2007; Naish et al., 2013; Nicholson et al., 2017; Decroix and Kalénine, 2018, 2019) or in a motor intention paradigm (Osiurak and Badets, 2014; Badets et al., 2017). This pattern was also found in another study in which participants were asked to decide whether word or picture stimuli of artifacts shared the same manipulation or the same function (Garcea and Mahon, 2012). Again, responses were faster for function judgments than for manipulation judgments. More direct evidence for the aforementioned exploratory pattern also comes from two recent eye-tracking studies, which have demonstrated that participants look first at the active part of artifacts and then at the manipulative part (Federico and Brandimonte, 2019, 2020). However, such a manipulative pattern does not suggest a motoric automatism generated by the mere observation of artifacts (i.e., the automatic activation hypothesis). Indeed, when the artifact shown is thematically inconsistent with the object presented (e.g., a bottle and a peaked cap), fixations are longer on the active part, indicating that people need to generate a potential mechanical action with the artifact and the object before engaging on the manipulative aspect. Crucially, the same non-manipulative visual-attentional pattern can be obtained by using an explicit non-motoric task (Federico and Brandimonte, 2020). Specifically, when people look at object-artifact pairs with the aim of recognizing them (i.e., a yes-no recognition paradigm), the artifact's active part receives more fixations, irrespective of thematic consistency. Taken together, these results highlight how people tend to use first the information needed to understand the mechanical action (involving the active part of the artifact), and then, when they are going to use an artifact, the information needed to select/plan the motor action involving the manipulative part.

Two other studies deserve mention (Kourtis and Vingerhoets, 2015; Kourtis et al., 2018). In both studies, participants were presented with pictures of an artifact for 1,000 s (Kourtis and Vingerhoets, 2015) or for 500–1,500 s (Kourtis et al., 2018), after which an arrow was overlaid on the artifact. Participants were asked to respond to make a left-hand response to a left-pointing arrow and vice versa. The artifact was tilted at an angle of 45° with its manipulative part pointing downward. In half the trials the active part was oriented toward the left (and the manipulative part toward the right) and vice versa for the other half. In both studies, they found that responses were faster when the arrow pointed to the active part of the artifact than to the manipulative part. This finding, which is the opposite of the orientation effect initially reported by Tucker and Ellis (1998), is consistent with many studies that have reported this opposite result, confirming its lack of robustness (see above). Kourtis et al. (2018) also observed that the congruency effect between the orientation of the active part of the artifact and the orientation of the arrow preferentially activated the left inferior parietal lobe (Talairach coordinates: *x* = −60, *y* = −34, *z* = 34). A potential interpretation of these findings is that they reflect the activation of specific motor representations of how to manipulate the artifact (i.e., functional affordances). However, another interpretation of these findings can be provided from recent advances.

The coordinates found by Kourtis et al. (2018) are very close to the ones reported by the meta-analysis conducted by Reynaud et al. (2016; i.e., *x* = −56, *y* = −31, *z* = 36) and corresponds to the brain area PF. In this meta-analysis, the activation of this brain area was associated with studies in which participants had to focus on the mechanical action between the active part of the tool and the object presented (see also Reynaud et al., 2019). Damage to this brain area is known to induce tool-use disorders (i.e., apraxia of tool use) in left brain-damaged patients not only when they have to use and select appropriate familiar tools/artifacts but also novel tools to solve mechanical problems (e.g., Goldenberg and Spatt, 2009; for review, see Osiurak et al., 2020). There is a consensus to consider that functional affordances cannot be useful to select or use novel tools because they are not associated with a specific function and, as a result, a specific manipulation (e.g., Buxbaum, 2017; Caruana and Cuccio, 2017). In other words, the brain area PF could be involved not in the selection/planning of motor actions associated withwith the manipulation of artifacts (i.e., functional affordances) but rather in the understanding of the mechanical actions that involves the artifact/tool—particularly, its active part—with an object, irrespective of the familiarity of the artifact/tool or the object. These findings question a potential functional-affordance-based interpretation about the activation of the brain area PF found in Kourtis et al. (2018). An alternative interpretation is that this activation reflects the involvement of a specific cognitive process concerned by the mechanical action that can be performed between the active part of the artifact and a potential object. In the next section, we will elaborate on this “specific cognitive process,” that is, technical reasoning.

## The Technical Reasoning Hypothesis

The automatic activation hypothesis of functional affordances is appealing and is fully consistent with the current widespread embodied cognition approach. This hypothesis is nevertheless subject to some theoretical and empirical limitations, which question its validity. More specifically, it does not predict the existence of a cascade mechanism (First, functional part ⇒ conceptual level; then, manipulative part ⇒ sensorimotor level.) Interestingly, this cascade mechanism is a key aspect of the technical reasoning hypothesis, which is akin to the classical amodal approach (Figure 1C; Osiurak et al., 2010; for a similar view, see also Goldenberg, 2013; Osiurak and Badets, 2016; Osiurak and Heinke, 2018). Technical reasoning can be broadly defined as the ability to reason about physical object properties. This nonverbal reasoning is both causal (i.e., predicting the effects on the environment of tool-use/making or construction actions) and analogical (i.e., transfer of what is understood from one situation to another). The technical-reasoning hypothesis assumes that people use tools—but also make them or build constructions—to solve physical problems in their everyday life (e.g, how to hang a picture on a wall; how to cut bread). In this respect, it diverges from most approaches to tool use in considering that tool use and the underlying cognitive processes are driven by the need to solve physical problems. To solve these problems, individuals first generate appropriate mechanical actions (e.g., cutting, lever) through technical reasoning. Then, they select the appropriate motor actions via the motor-control system in order to realize the mechanical action generated through technical reasoning. This cascade mechanism can be reiterated if necessary, for instance, when the mechanical action generated does not work, which can require the generation of another mechanical action or the selection of most appropriate tools.

The technical reasoning hypothesis has been mainly developed from studies in which participants are asked to actually use tools with objects contrary to most of the studies discussed here. According to this hypothesis, technical reasoning is not viewed as involved in semantic processing, such as when someone has to categorize an object as natural or manmade or to name it. Nevertheless, this hypothesis does not assume that people need to be in the presence of tools and objects to reason about the potential mechanical actions that can be performed with them. This reasoning can be done offline, for instance, when someone reasons about which nail of the workshop can be useful to hang a paint a picture on the wall of the living room. In this respect, it can be considered that the presentation of pictures of artifacts on a computer screen can lead participants to initiate such reasoning. Even if this hypothesis assumes a cascade mechanism that seems to be consistent with the findings discussed here, it is noteworthy that a significant theoretical effort is needed to explain how technical reasoning can interfere in recognition tasks (for discussion of this limitation, see Buxbaum, 2017). The fact remains that the technical reasoning hypothesis offers a viable alternative to the automatic activation hypothesis of functional affordances to explain how humans conceive the different objects of the environment as potential tools. Having said this, we believe it is noteworthy that a theoretical effort remains to be made to reconcile the technical reasoning hypothesis with the automatic activation hypothesis of functional affordances. Indeed, whereas the initial goal of the former is to explain how humans select and use tools to solve physical problems (i.e., focus on the mechanical action between the tool and object in a context of problem solving), the latter has been developed to account for how humans acquire knowledge about tools based on their sensorimotor interactions with them (i.e., focus on the motor action between the tool and the hand in a context of knowledge acquisition).

## Author Contributions

FO developed the article concept and drafted the manuscript. GF, MB, ER, and ML provided critical revisions. All authors contributed to the article and approved the submitted version.

## Conflict of Interest

The authors declare that the research was conducted in the absence of any commercial or financial relationships that could be construed as a potential conflict of interest.

## Footnotes

^1^When someone uses a tool with an object, two kinds of relationships can be described, namely, tool-hand relationships and tool-object relationships (Goldenberg, 2013). We will use the term “motor action” to refer to the bodily action performed by the user that involves a tool-hand relationship. Motor actions concern preferentially the manipulative part of the tool/artifact. Note that motor actions are not limited to the use of tools but can also concern non-tool use actions (e.g., grasping, reaching). In addition, we will use the term “mechanical action” to refer to the physical action performed by the tool with the object (i.e., tool-object relationships). Mechanical actions concern preferentially the active part of the tool/artifact.

^2^The term “functional way” refers to the use associated with the usual function of the artifact, that is, the purpose for which the artifact was made (e.g., pounding a nail for a hammer, driving a screw for a screwdriver).

^3^The terms “gesture engram” (Buxbaum, 2001), “visuokinesthetic motor engram” (Heilman et al., 1982), “spatial-temporal movement representation” (Heilman and Watson, 2008), “manipulation knowledge” (Bach et al., 2014; van Elk et al., 2014) or “representation of motor programs for acquired tool use skills” (Johnson-Frey et al., 2005), among others, have also been used as synonyms of functional affordance. Nevertheless, even if the terminology varies, the concept remains similar and differs from the initial definition of affordance proposed by Gibson (1986; for a review about the multitude of meanings of the term affordance, see Osiurak et al., 2017).

^4^Here, we mean by “embodied cognition approach” any embodied cognition approach assuming that humans possess knowledge that is distributed in the different sensorimotor systems (e.g., Barsalou, 2008, 2009; Thill et al., 2013; van Elk et al., 2014; Buxbaum, 2017). In other words, this does not concern the ecological approach to perception initiated by Gibson (1986), that is, a nonrepresentational approach, which is sometimes assimilated to the embodied cognition approach.

^5^Some hybrid amodal-embodied attempts have been proposed on the grounds that extreme views have little empirical support (e.g., Chatterjee, 2010; Dove, 2011; Borghi et al., 2017; see also Meteyard et al., 2012; Matheson et al., 2015; Michel, in press). These hybrid attempts have been made in the field of linguistic/semantic processing (i.e., when applied to tools, naming a tool, or knowing its usual function or context of use). However, in the field of “tool use” (i.e., actual tool use or pantomime of use), most of the models have adopted a more extreme version of the embodied cognition approach, in suggesting that tool use is based on the activation of sensorimotor representations about manipulation, that is, functional affordances (e.g., Buxbaum and Kalénine, 2010; Thill et al., 2013; van Elk et al., 2014). This is also true for early models, even if they were not labeled as embodied (Heilman et al., 1982; Rothi et al., 1991; Cubelli et al., 2000; Buxbaum, 2001).
